# Summit Discusses Public Health Implications of Fracking

**DOI:** 10.1289/ehp.121-a15

**Published:** 2013-01-01

**Authors:** Tanya Tillett

**Affiliations:** Tanya Tillett, MA, of Durham, NC, is a staff writer/editor for *EHP*. She has been on the *EHP* staff since 2000 and has represented the journal at national and international conferences.

In the wake of the North Carolina state legislature’s July 2012 vote to legalize new oil and gas exploration,^1^ the state is poised to join the ranks of others already tapping into shale gas deposits with the help of hydraulic fracturing (fracking). But public and environmental health concerns, particularly those related to air emissions^2^ and drinking water safety and security,^3^^,^^4^^,^^5^ have sparked controversy in states such as Pennsylvania, where fracking is already being used to extract natural gas. At the fifth annual summit of the Research Triangle Environmental Health Collaborative, based in Research Triangle Park, North Carolina, stakeholders worked in three breakout groups over two days to recommend best practices for policy makers, the fracking industry, and community members in this and other states.^6^

In summer 2012 the U.S. Geological Survey released an assessment of undiscovered oil and gas resources in North Carolina,^7^ which state geologist Kenneth Taylor says equals about five years’ current consumption for the state. Gas extraction in North Carolina’s Sanford sub-basin—the most promising area for drilling—has been projected to provide an average of 387 jobs per year over seven years.^8^ Meanwhile, research on the potential human health effects of fracking remains in its early days.

The exposures workgroup cited needs for information on water availability, air quality standards (with guidelines for sampling and analysis), and potential ecosystem impacts, as well as for baseline health data about community members and gas workers. “North Carolina has the advantage of actually being able to do this since there is no prior history of oil and gas production,” says Scott Masten, a senior toxicologist in the National Toxicology Program Division of the National Institute of Environmental Health Sciences. A participant in the exposures workgroup, Masten also delivered a plenary speech on federal government activities related to fracking.

Potential health impact issues discussed included the need for comprehensive, ongoing health and demographic data collection, risk modeling, and public awareness campaigns, as well as a stable funding mechanism for all these endeavors. Discussion also centered on establishing best management practices for drillers (well standards, site design standards, inspections, etc.) to help safeguard public health.

Hope Taylor, executive director of the nonprofit Clean Water for North Carolina, says there are tens of thousands of private well users in each of the counties that could potentially be affected by gas extraction. “We simply do not know, even in deeper shale formations, all of the conditions that would be required to carry out such operations to prevent contamination,” she says. “In North Carolina’s very shallow and discontinuous shales, groundwater supplies would be at greater risk.”

Recommendations from the social impacts workgroup included identification of legal and physical impacts on landowners and others living near drilling locations, economic impacts to affected communities, and impacts to state and local infrastructure that would accompany the increased traffic, equipment, and activities associated with drilling. Hope Taylor pointed out that contractors, investors, pipeline companies, and large landowners would receive most of the economic benefits of development, whereas communities would bear most of the externalized costs related to noise, air emissions, potential drinking well contamination, traffic, and community disruption—a disconnect she said must be considered in impact analyses.

According to Emily McGraw, state maintenance operations engineer with the North Carolina Department of Transportation, who participated in a state panel discussion at the summit, the Department of Transportation in Pennsylvania has been helpful with the infrastructural analysis process, providing guidance on permitting and bonding issues for hauling on rural roadways. “The biggest impact will be the weight of the loads coming in . . . [leading to] increased traffic and possible structural deterioration,” she says.

Bernard Goldstein, professor emeritus of the Department of Environmental and Occupational Health at the University of Pittsburgh, says planning initiatives such as this summit and the recent Institute of Medicine Roundtable on Shale Gas Extraction^9^ are beneficial for states that may pursue fracking, and that the industry is getting better at dealing with environmental issues. Goldstein, who did not attend the summit, coauthored a recent *EHP* commentary on the current lack of a prominent role for the environmental public health community on the advisory boards making recommendations concerning drilling for natural gas.^10^ He says, “Technology that’s getting better over time has helped drillers do a better job of dealing with what comes up from underground—chemicals, brine, arsenic, radioactivity.”

Dennis Devlin, senior environmental health advisor for Exxon Mobil Corporation, agrees. “To ensure that the economic benefits of unconventional gas development continue, our industry is committed to continue properly managing the risks involved with energy production,” he says. “This means meeting the highest standards of well design and integrity. We have rigorous standards in place to monitor and maintain wells after drilling is complete.”

Given all the considerations that came out of the summit, would hydraulic fracturing be a feasible enterprise in the state of North Carolina? Yes, says state geologist Taylor, but feasibility would depend on satisfactorily addressing a host of key issues: the implementation of guidelines for hydraulic fracturing, adequate standards for gas well construction, rules on the reuse of wastewater, and the management of drilling waste.

Goldstein agrees, and advises policy makers and stakeholders in North Carolina to practice caution, saying it would be best not to advance without more safety and technological advancements to protect public health. Rob Jackson, Nicholas Chair of Global Environmental Change at Duke University’s Nicholas School of the Environment and coauthor of a recent white paper on research and policy recommendations for fracking,^11^ summed it up for participants: “The best lesson we can learn from Pennsylvania is ‘don’t hurry.’ We need to make sure that we have strong rules and regulations in place, the resources for enough inspectors to do their job, and a commitment to keeping track of everything, from wastewater disposal to the chemical composition of hydraulic fracturing fluids.”
